# Enhanced Deep-Inspiration Breath Hold Superior to High-Frequency Percussive Ventilation for Respiratory Motion Mitigation: A Physiology-Driven, MRI-Guided Assessment Toward Optimized Lung Cancer Treatment With Proton Therapy

**DOI:** 10.3389/fonc.2021.621350

**Published:** 2021-04-29

**Authors:** Frank Emert, John Missimer, Philipp A. Eichenberger, Marc Walser, Celina Gmür, Antony J. Lomax, Damien C. Weber, Christina M. Spengler

**Affiliations:** ^1^Center for Proton Therapy, Paul Scherrer Institute (PSI), Villigen, Switzerland; ^2^Exercise Physiology Lab, Department of Health Sciences and Technology, Institute of Human Movement Sciences and Sport, ETH Zurich, Zurich, Switzerland; ^3^Department of Physics, ETH Zurich, Zurich, Switzerland; ^4^Department of Radiation Oncology, University Hospital Zurich, Zurich, Switzerland; ^5^Department of Radiation Oncology, University Hospital Bern, Bern, Switzerland; ^6^Zurich Center for Integrative Human Physiology (ZIHP), University of Zurich, Zurich, Switzerland

**Keywords:** breath hold, enhanced DIBH, HFPV, proton therapy, lung cancer, motion mitigation, MRI, lung volume

## Abstract

**Background:** To safely treat lung tumors using particle radiation therapy (PRT), motion-mitigation strategies are of critical importance to ensure precise irradiation. Therefore, we compared applicability, effectiveness, reproducibility, and subjects' acceptance of enhanced deep-inspiration breath hold (eDIBH) with high-frequency percussive ventilation (HFPV) by MRI assessment within 1 month.

**Methods:** Twenty-one healthy subjects (12 males/9 females; age: 49.5 ± 5.8 years; BMI: 24.7 ± 3.3 kg/m^−2^) performed two 1.5 T MRI scans in four visits at weekly intervals under eDIBH and HFPV conditions, accompanied by daily, home-based breath-hold training and spirometric assessments over a 3-week period. eDIBH consisted of 8-min 100% O_2_ breathing (3 min resting ventilation, 5 min controlled hyperventilation) prior to breath hold. HFPV was set at 200–250 pulses min^−1^ and 0.8–1.2 bar. Subjects' acceptance and preference were evaluated by questionnaire. To quantify inter- and intrafractional changes, a lung distance metric representing lung topography was computed for 10 reference points: a motion-invariant spinal cord and nine lung structure contours (LSCs: apex, carina, diaphragm, and six vessels as tumor surrogates distributed equally across the lung). To parameterize individual LSC localizability, measures of their spatial variabilities were introduced and lung volumes calculated by automated MRI analysis.

**Results:** eDIBH increased breath-hold duration by > 100% up to 173 ± 73 s at visit 1, and to 217 ± 67 s after 3 weeks of home-based training at visit 4 (*p* < 0.001). Measures of vital capacity and lung volume remained constant over the 3-week period. Two vessels in the lower lung segment and the diaphragm yielded a two- to threefold improved positional stability with eDIBH, whereby absolute distance variability was significantly smaller for five LSCs; ≥70% of subjects showed significantly better intrafractional lung motion mitigation under reproducible conditions with eDIBH compared with HFPV with smaller ranges most apparent in the anterior-posterior and cranial-caudal directions. Approximately 80% of subjects preferred eDIBH over HFPV, with “less discomfort” named as most frequent reason.

**Conclusions:** Both, eDIBH, and HFPV were well-tolerated. eDIBH duration was long enough to allow for potential PRT. Variability in lung volume was smaller and position of lung structures more precise with eDIBH. Subjects preferred eDIBH over HFPV. Thus, eDIBH is a very promising tool for lung tumor therapy with PRT, and further investigation of its applicability in patients is warranted.

## Introduction

The safe, accurate, and effective delivery of a highly conformal dose to the tumor while sparing adjacent healthy tissues represents the central challenge in the delivery of external beam radiation therapy (1–4). A fundamental advantage of particle radiation therapy (PRT) is the steep dose gradient at the distal edge, which allows protons and carbon ions to deliver their therapeutic dose with a precisely defined, energy-dependent finite range distribution (4, 5). This highly sophisticated level of spatial precision requires exact knowledge of the tumor in space and time during the entire treatment, especially in the presence of motion (6, 7). This also includes the time-dependent distribution of the materials along the particle trajectory, since the materials' electron densities have a significant influence on particle range.

With respect to proton therapy for mobile tumors, e.g., lung tumors, using pencil beam scanning (PBS-PT), the following factors are the major challenges to delivering the intended dose distribution (7–10): (i) target failures due to interfractional tumor changes regarding position, shape, and size; (ii) dose blurring due to interfractional changes in the patient's anatomy due to density variations along the beam path, and (iii) intrafractional interplay between the dynamics of the beam and the motion of anatomical structures due to respiration, heartbeat, gastrointestinal peristalsis, and inertial organ relaxation. The problem of treating lung tumors with PBS-PT was already discussed in 1992 (11), and the delivery of such treatments has gradually evolved since (12, 13). All main components of proton therapy workflows are now time dependent, including motion analysis and modeling, multimodal imaging, contouring, treatment planning, dose delivery techniques, and integrated patient monitoring (14–17). So-called 4D treatment strategies and motion management concepts continue to be developed to cope with this temporal dependence and to meet the corresponding specific challenges of proton range uncertainties (18–21).

In practice, these approaches can be divided into techniques which either manipulate the treatment beam (e.g., tracking, gating, rescanning, robust optimization, etc.,) (22–25) or those that mitigate target motion (e.g., compression, breath hold, ventilation) (26, 27). Although, applications of combinations of these can be effective, the selected treatment approach generally depends on individual patient and tumor conditions (28), as well as on site-specific irradiation capabilities (17).

Of the different patient-assisted motion mitigation techniques available, suppression of ventilation via active or passive breath holding seems to be among the most promising (17). Important in this context is the duration of motion suppression and its influence on the stability of lung structure and volumes. For example, considering relationships between tumor volumes and field application durations for different rescanning scenarios based on the beam delivery characteristics of, e.g., Gantry-2 at our institution, realistic rescanning factors are 0–4 with a duration of 45–90 s for a single field irradiation for tumor volumes up to 1 L. Therefore, with the goal of one breath hold per irradiation field, breath-hold durations of more than 60–90 s are desirable. This is longer than the length of unassisted, voluntary breath holds that typically range between 30 and 70 s and are typically associated with chest wall movements when subjects approach the point of termination. Without training, a longer breath-hold duration can only be achieved either by physiological interventions prior to an active deep-inspiration breath hold (DIBH) or via passive “breath holding” using, for example, high-frequency mechanical ventilation, e.g., high-frequency percussive ventilation (HFPV) (27).

For active breath holding, however, the challenges are manifold. First, lung volume and chest wall positions need to be reproducible in relation to the planning CT (the basis for treatment). Since the largest breath-hold duration can be achieved with the largest lung volume, subjects must hold their breath at maximal inspiration, i.e., at total lung capacity (29, 30). However, measures of lung volumes are known to vary in response to a person's experience with performing this specific inspiratory maneuver. This implies that on each measurement day, several full inspirations need to be performed until stable total lung capacity values are reached (31, 32). Second, breath holds may be more difficult in supine position, as active breath hold in daily life is used for stabilizing the trunk during lifting heavy objects or balancing, in contrast to the relaxed supine position. Furthermore, additional weight with abdominal obesity may cause further objective or subjective problems. Third, coughing is one of the most common symptoms of lung cancer. The majority of lung cancer patients are current or former smokers, potentially also suffering from chronic obstructive pulmonary disease (COPD) which is also accompanied by frequent coughing due to bronchitis. Tussive irritation may, in turn, lead to early termination of breath holding, a problem most likely to be mitigated through medication.

Perhaps the most limiting factor of breath-hold duration, however, is the so-called break-point, where perception of intolerable air hunger urges subjects to take a new breath. This depends on the interplay of multiple factors. First, rises in arterial CO_2_-partial pressure (P_a_CO_2_) stimulate the respiratory motor output via chemoreceptors, giving rise to air hunger. Second, the ability of the subject to suppress respiratory motor output and/or tolerate the lack of rhythmical input from lung and chest wall stretch receptors (33, 34) also limit breath-hold duration. Third, also decreases in O_2_-partial pressure (P_a_O_2_) that stimulate respiratory motor output *via* chemoreceptors also rise to air hunger—an effect important mainly during breath holding after prior hyperventilation. Importantly, the O_2_ available depends on lung size that decreases with age, is lower in women, and is often reduced by tumor tissue itself. Also, during extended breath holds, lung volume can decrease, since less CO_2_ is produced than the O_2_ that is consumed. Last, the psychological state of a person also contributes to the level of perceived air hunger (35). Thus, an anxious person may reach the break-point earlier.

On the other hand, it has also been demonstrated that breath-hold duration can be extended using a variety of techniques. For instance, hyperventilation prior to breath holding decreases the starting P_a_CO_2_ and thus leads to delayed chemoreceptor and ventilatory stimulation. As a small risk for passing out exists with hyperventilation, however, the level of P_a_CO_2_ needs to be controlled for patient safety. Training of the subjects, by which they become familiar to the suppression of the automatic respiratory motor output and to the lack of stretch receptor input, has also been shown to extend breath-hold duration (36). Finally, extended breath-hold duration has been demonstrated by breathing a gas mixture with increased P_a_O_2_ leading to delayed chemoreceptor and thus ventilatory stimulation when P_a_O_2_ decreases (37, 38).

Similarly, passive breath holding via HFPV is not without challenges for conscious subjects. First, patients need to “hold” a mouthpiece tightly in their mouth while fully relaxing muscles of the chest and the diaphragm. It is yet unknown whether this technique can be trained by repeated application. Normally, a patient in need of HFPV is sedated or unconscious in the intensive care unit. Second, the pressure applied, and the level of muscle relaxation, greatly affects pulmonary compliance and therefore lung volume at a given pressure, thus, potentially affecting the location of anatomical structures. Finally, HFPV may induce motion artifacts due to the vibrating nature of this type of ventilation. Although, expected to be in the millimeter range, this could be of considerable importance for its application in proton therapy. Despite these limitations, as both active and passive breath-hold techniques can substantially reduce motion amplitudes, they could be of considerable interest in radio- and proton therapy as effective motion mitigation techniques.

The aim of the present study therefore, was to compare, in healthy volunteers, intrasession, and intersession variability of lung volumes, position of anatomical lung structures, and breath-hold durations between physiologically modified oxygen-enhanced DIBH (eDIBH) and HFPV over the duration of 3 weeks in order to simulate fractionated radiotherapy treatment regimes. Longitudinal breath-hold duration, lung volume measurement, and magnetic resonance imaging (MRI) were used to assess variability in volunteers performing daily breath-hold training.

## Materials and Methods

### Participants

Twenty-one healthy subjects (12 males, 9 females) participated in the study (Table 1). Inclusion criteria were absence of physical and mental impairment or disease, age ≥40 years, and ability to give informed consent by signature. Subjects were excluded, if they had contraindications to MRI procedures, i.e., non-MRI-suitable electronic and metal implants or claustrophobia, impaired lung function, acute or chronic disease, known or suspected non-compliance, drug or alcohol abuse, inability to follow the procedures of the study (e.g., due to language problems, psychological disorders, or dementia of the participant), presence of any psychological or sociological condition potentially hampering compliance with the study protocol or, for women, pregnancy, or breastfeeding. Subjects were informed about all procedures, and all devices were shown and explained before subjects signed an informed consent prior to the first data collection. The study was approved by the Cantonal Ethics Committee North West and Central Switzerland (BASEC-ID: 2018-01295; Clinical trial number NCT03669341).

**Table 1:** Demographic data of subject cohort.

**Parameter**	**Males**	**Females**	***p***
	**(*n* = 12)**	**(*n* = 9)**	
Age [years]	50.7 ± 6.4	47.9 ± 4.6	0.496
Height [cm]	183.2 ± 5.3	167.8 ± 5.0	<0.001
Weight [kg]	89.0 ± 9.5	62.9 ± 9.5	<0.001
BMI [kg·m^−2^]	26.5 ± 2.4	22.3 ± 2.7	0.003

*Data presented as mean ± SD. BMI: Body mass index*.

### Study Protocol

**Figure 1:**
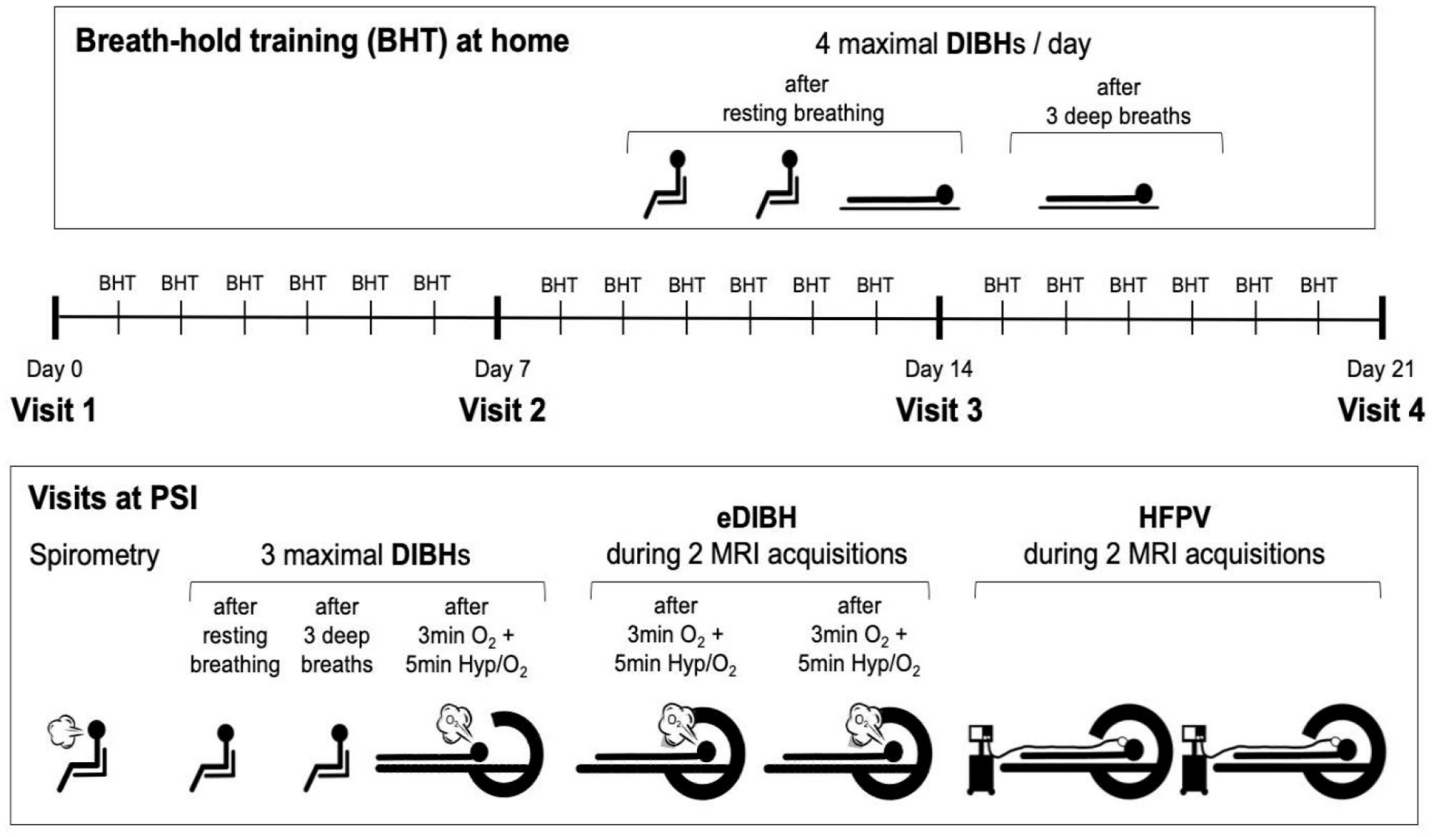
Study protocol. Visits at the Paul Scherrer Institute (PSI) were interspersed by 6 days of home-based breath-hold training with four deep-inspiration breath holds (DIBHs) of maximal duration, two in sitting and two in supine position. The first three breath holds were performed out of resting breathing, the fourth after three deep breaths. At visits, first spirometry was performed, followed by two DIBHs of maximal duration in sitting position, the second one performed after three deep breaths. The third breath hold prior to magnetic resonance image (MRI) acquisitions was performed after ≥8 min of breathing 100% O_2_ with hyperventilation (at an end-tidal CO_2_-partial pressure of 20 mmHg) for the last 5 min prior to this maximal enhanced DIBH (eDIBH). This procedure was immediately followed by moving the subject into the MR scanner, initiating the eDIBH-procedure prior to each of two consecutive MRI acquisitions and maintaining it during image acquisitions. After moving the subject out of the MR scanner and installing the high-frequency percussion ventilator (HFPV), the subject was moved into the scanner ring again for two consecutive MRIs under HFPV conditions.

Subjects visited the Center for Proton Therapy at the Paul Scherrer Institute (PSI, Villigen, Switzerland) for MRI acquisitions on four different occasions. Visits were interspersed by 6 days of home-based breath-hold training. A schematic of the study protocol is displayed in Figure 1. In addition, a fifth visit was arranged for four selected subjects who demonstrated long breath-hold durations whereby three MRI acquisitions were performed within the same breath hold.

At the first visit, subjects were thoroughly informed about all details of the study procedures, including a demonstration of all testing equipment. After signing the informed consent form, a questionnaire-based assessment regarding cardiovascular risk factors and exclusion criteria was performed. Then, subjects were briefly asked about activity, nutrition, and sleep over the past 24 h, which they were requested to keep as similar as possible before each of the following visits to assure a similar metabolic and psychological status.

At all visits, after checking the current health status as well as activity, nutrition, and sleep over the past 24 h, lung function was assessed by performing forced spirometry. This served to exclude a potentially undiagnosed lung disease affecting lung volume and/or respiratory mechanics and to achieve maximal lung volumes for breath holding because forced spirometry, yielding FVC, requires repeated maximal inspiration until similar volumes are reached (31).

Thereafter, and as with home training, two DIBH of maximal duration were performed in sitting position, the first out of resting breathing, the second after three deep breaths (to decrease P_a_CO_2_ slightly and prolong breath-hold duration).

Next, the subjects were transferred to the MR scanner and positioned on the couch in supine position according to a positioning under treatment. Measurement devices for the assessment of heart rate (HR) and peripheral blood oxygen saturation (SpO_2_) were used to assure subject's safety throughout the procedure. Also, respiratory CO_2_ concentration was measured to control the level of hyperventilation. Subjects also were fitted with a face mask for delivery of 100% O_2_ during hyperventilation prior to breath-holding in the MR scanner.

One enhanced DIBH (eDIBH) procedure was then performed to determine the maximum breath-hold duration. Thereafter, subjects were moved by the MR couch into the scanner ring head first and a laser alignment (for reproducible interfractional positioning between weekly visits including wing board settings) followed. During two immediately consecutive MRI acquisitions performed to simulate intrafractional treatment conditions with multiple fields, subjects were asked to perform two separate eDIBHs, one for each scan. Afterwards, subjects were moved out of the MR scanner to return to resting breathing and allowed to move (still on the couch).

Subsequently, details of the HFPV procedure were explained again and subjects were connected to the HFPV device. A first HFPV trial was performed outside the MR scanner for familiarization purposes, to adjust the HFPV settings accordingly and to test whether the tolerated HFPV duration was sufficient for a single MRI acquisition. Subjects were then repositioned and moved back into the MR scanner, where two consecutive MRI acquisitions were performed, in analogy to the two eDIBHs.

At the end of the first session, subjects additionally received instructions on performance and recording of their daily, home-based breath-hold training which was recorded in a personal logbook (see Section **Training and Measurements at Home** for details).

### Measurements and Interventions During Each Visit

#### Lung Function Measurement

Forced spirometry was performed to assess FVC as well as maximal inspiratory and expiratory flow rates via a hand-held spirometer (Spirobank, MIR, Rome, Italy). Measurements were performed according to criteria of the American Thoracic Society and the European Respiratory Society (31). An adequate test required a minimum of three acceptable maneuvers and meeting the reproducibility criteria according to the ATS/ERS statement. If these criteria were not met, additional trials were performed until criteria were met but no more than eight maneuvers were performed.

#### Motion Suppression Techniques

The eDIBH procedure was performed as follows: In resting, supine position, subjects breathed 100% O_2_ via face mask for at least 8 min with (i) 3 min resting breathing followed by (ii) controlled hyperventilation where subjects were coached to perform deep inspirations and expirations such that a P_ET_CO_2_ of 20 mmHg (2.67 kPa) was maintained for 5 min, followed by (iii) a maximal deep inspiration, which was sustained for as long as possible. A second and third eDIBH was performed during the following MR scans, where eDIBH was maintained during image acquisition for 70 s.

HFPV is based on the administration of small volumes of air, so-called percussions, with adjustable pressures and frequencies. These percussions can replace spontaneous ventilation allowing prolonged apnea-like suppression of respiratory motion while maintaining adequate oxygen diffusion and CO_2_ removal.

The HFPV procedure was performed using a jet ventilator (Monsoon^®^ Jet Ventilation, ACUTRONIC Medical Systems, Hirzel, Switzerland), positioned in the MR control room (outside the magnetic scanner region). The ventilator was connected by an 8-m pressure tube to an open circuit breathing-adapter (Phasitron^®^, Percussionaire Suisse, Ardon, Switzerland) that subjects held in their mouth via mouthpiece with a nose clip in place. The familiarization trial outside the MR scanner was performed using 1.0-bar pressure pulses at a frequency of 250 pulses min^−1^. Individual adjustments of pressure and frequency were made according to subjective comfort and the subjects' ability to relax while being passively ventilated. During the MRI acquisitions, individualized pressure pulses ranged from 0.8 to 1.2 bar with a frequencies of 225–250 pulses min^−1^.

#### MR Positioning and Image Acquisition

Subjects were immobilized on the MR couch using (i) a removable wing board attached to it with a central head support and adjustable fixation rods, which were held by both hands with arms supported overhead, and (ii) a knee support without additional fixation devices.

For all image acquisitions in the MR scanner (MAGNETOM Aera, 1.5 T, Siemens Healthcare AG, Zurich, Switzerland) a T2-weighted, 2D-steady-state free precision (SSFP) sequence was used. Voxel spacing was 0.7617 by 0.7617 mm^2^ in plane with a plane separation of 2.2 mm; the reconstructed image plane consisted of an array of 512 × 512 pixels. Approximately 100 coronal 2D image planes were acquired in about 70 s.

#### Visit 5 for Selected Subjects

Four subjects that were capable of maintaining eDIBH for at least 4 min were recruited for an additional session to assess changes in lung volume during prolonged breath-holding. During this fifth visit, only the eDIBH procedure was performed. The visit consisted of two separate MRI acquisitions, each with three consecutive MR sequences, and each performed over a period of 210 s during a single eDIBH.

#### Subjective Assessment of Interventions

At the end of visit 4, acceptance of breath-hold training at home and levels of “comfort/tolerability” of eDIBH or HFPV procedures during the MRI acquisition were assessed using visual analog scales with the anchors “feasible”/“not feasible” and “well-tolerable”/“not tolerable.” Finally, the subjects were asked which of the two techniques for respiratory motion mitigation they would prefer as a patient.

### Training and Measurements at Home

Home-based breath-hold training consisted of four maximal DIBHs per day for a total of 18 days in order to get used to these respiratory maneuvers including full inspirations. The first and second DIBH was performed in sitting position, out of resting breathing as this is more comfortable and to allow comparison with the same breath-hold technique in lying position. The third and fourth DIBH were performed in lying position, with the first of these two also starting from resting breathing (to be compared with sitting) while the second was initiated after three deep inspirations and expirations to show the subject that “hyperventilation” increases breath-hold duration and to get them used to long-duration breath holds. Breath-hold durations were recorded by the subject in a logbook and correct recording was double-checked at each lab visit.

### Data Analysis and Statistics

Anthropometric data were compared between sexes using the Mann-Whitney *U* test.

#### Breath-Hold Duration, Forced Vital Capacity, and Subjective Assessment

In order to evaluate the effects of sex and repeated visits, breath-hold durations as well as forced vital capacities were analyzed using two-way analysis of variance (ANOVA) with repeated measures and Bonferroni corrections for *post-hoc* analyses. If data were not normally distributed, a related-sample Friedman test was performed and the Wilcoxon signed rank test used for *post-hoc* analysis. Effects of breath-hold methods and sex in time-averaged home training (18 days average), visit (four visits average), and subjective data were analyzed using paired *t*-tests or Wilcoxon matched-pairs signed rank test. For correlation analysis between breath hold and spirometry variables, Pearson's correlation coefficient was calculated if data were normally distributed and Spearman's correlation coefficient if data were not normally distributed. One subject was excluded from all breath-hold analyses due to incomplete data. GraphPad Prism 2019 (GraphPad Software, La Jolla California, USA) was used for statistical analysis and data presentation.

#### Lung Volume Analysis

Based on the repeated MRI acquisitions using both eDIBH and HFPV inter- and intrafractional lung volumes and displacements have been determined and compared. Total lung volumes were determined from each acquired MRI using an automatic segmentation program developed in-house. Using functions provided by the Image Processing Toolbox of MatLab (R2018b, The MathWorks, Inc., Natick, Massachusetts, USA), the program imported and saved image data in DICOM format. The algorithm first processed the image volume as suggested in the step “Mark the Foreground Objects” in the MatLab documentation section: “Marker-Controlled Watershed Segmentation.” A voxel threshold was selected by an adaptive algorithm for each image plane and the segmentation effected using the functions bwlabel.m and regionprops.m. The final step in the algorithm was the application of the 3D-clustering program, spm_cluster.m of the statistical parametric mapping package SPM12 (https://www.fil.ion.ucl.ac.uk/spm/software/spm12/). Two-way unbalanced ANOVAs evaluated the effects of visit, motion-mitigation method, and sex. For the four subjects studied for lung volume decreases during the acquisitions (fifth visit), the volumes were normalized by the initial volumes to yield a fractional decrease during the acquisition.

#### Measuring Intra- and Interfractional Lung Displacements

To simulate potential intra- and interfractional variations in lung volume and form as part of a radiotherapy treatment, in this study, it has been assumed that the breath-hold duration of a patient with eDIBH is sufficient for the complete irradiation of one field. That is, each single MRI reflects lung position and shape for an individual irradiation field, with each fourfold weekly repetition of two consecutive MRI sequences then representing four irradiation fractions, each consisting of two fields. As such, variations between MRIs in the same session represent inter-field variations, whereas, variations between sessions represent potential interfraction variations. In addition, variations of intrafield conditions, i.e., intrabreath-hold changes in lung volume, were evaluated by the acquisition of the three consecutive MRIs within one eDIBH in selected subjects.

**Figure 2:**
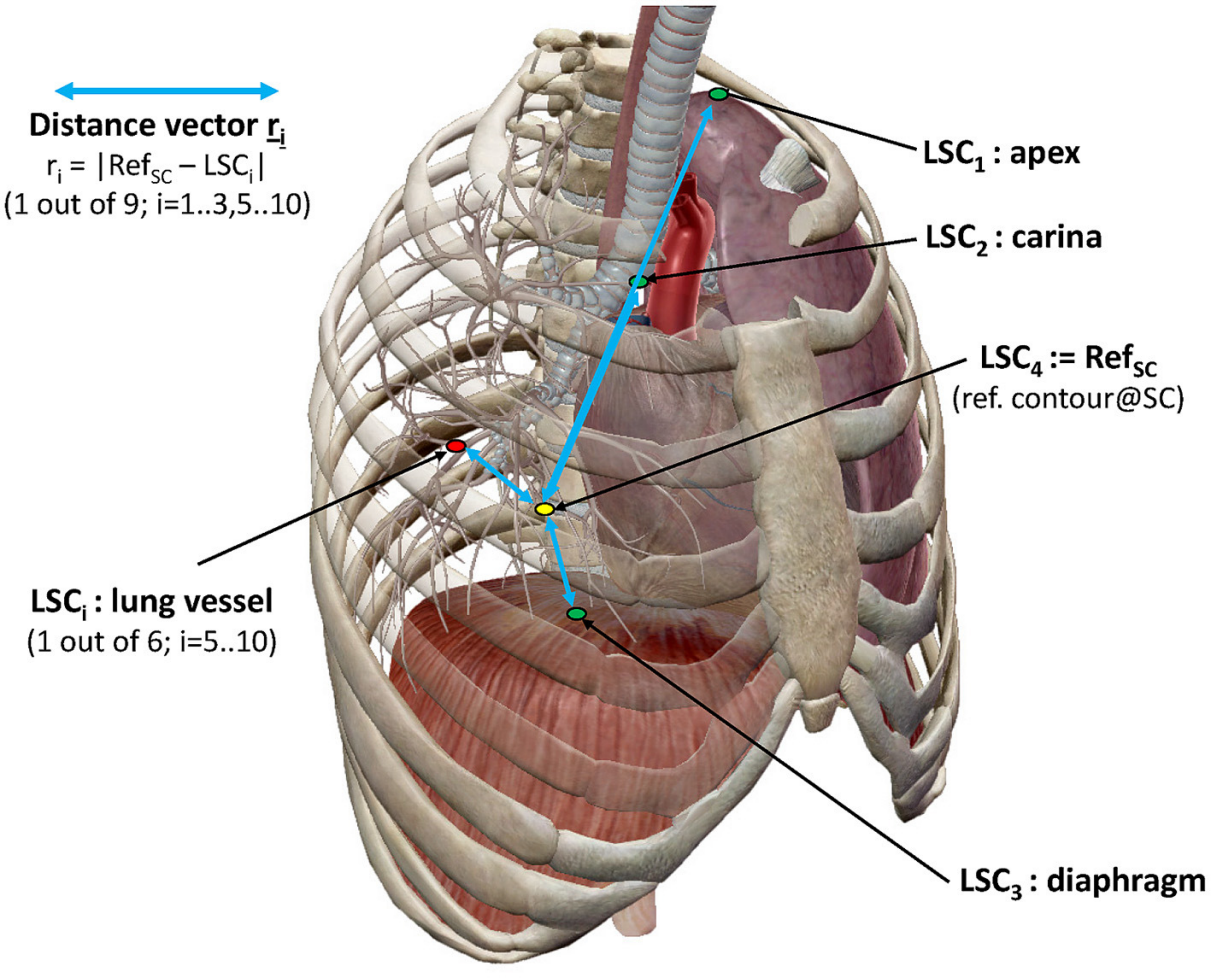
Representation of lung structure metric with selected LSCs. LSC distribution as described in the text (image courtesy of Visible Body^®^).

In order to investigate the spatial variation of key anatomical lung structures (LSCs) over therapy sessions, a reference point was selected (Ref_SC_ = LSC_4_) which does not move during respiration in the supine position. The chosen reference point was a prominent paravertebral part of one of the posterior intercostal veins on the level of the middle thoracic vertebral column (T4–T8). As shown in Figure 2, the selected anatomical structures subject to movement were (i) the ribs in the apex areas of the lung (LSC_1_), (ii) the carina tracheae (LSC_2_), (iii) the diaphragm (LSC_3_), and (iv) the branching points of six specific lung vessels representing hypothetic intrapulmonal tumor locations (LSC_5_-LSC_10_). The distribution of the six vessels observed an even division in the craniocaudal direction. With an extension from the apex to the costodiaphragmatic cavity, projections on the junctions of the T5/T6 vertebras and T9/T10 were selected, which defined three segments of approximately equal length. On each side of a segment were then located two vessels. Using Velocity^®^, trained medical assistants located the reference point and anatomical structures manually; two of the authors (FE and MW) checked the locations. The set of difference vectors **r**_**i**_ between the locations, Ref_SC_ and LSC_i_, is denoted as an intrapulmonary lung structure metric. Using these metrics, two analyses have been performed.

First, the radial distance $r_{\text{i}}={\underline{|r}}_{\text{i}}\underline{|}$ of the distance vector **r**_**i**_, was determined for each MRI acquisition. From the sets of eight MRIs (two scans at four visits) acquired for each subject, the mean radial distances of each lung structure *μ*(*r*_i_, *M*) and their standard deviations *σ*(*r*_i_, *M*) were determined for each method *M*: eDIBH or HFPV. The ratios *σ*(*r*_i_, eDIBH)/μ(*r*_i_, eDIBH) and *σ*(*r*_i_, HFPV)/*μ*(*r*_i_, HFPV) yielded fractional variations, for which medians, maxima, and distributions of ranks according to the Wilcoxon signed rank test could be compared. In addition, the logarithm of the ratio of fractional variations.

$$\begin{array}{l} \gamma =\log[\frac{\frac{\sigma \left(r_{\text{i}},\text{eDIBH}\right)}{\mu \left(r_{\text{i}},\text{eDIBH}\right)}}{\frac{\sigma \left(r_{\text{i}},\text{HFPV}\right)}{\mu \left(r_{\text{i}},\text{HFPV}\right)}}]\;. \end{array}$$

of the two methods provided a comparison of methods for each subject and lung structure.

**Figure 3:**
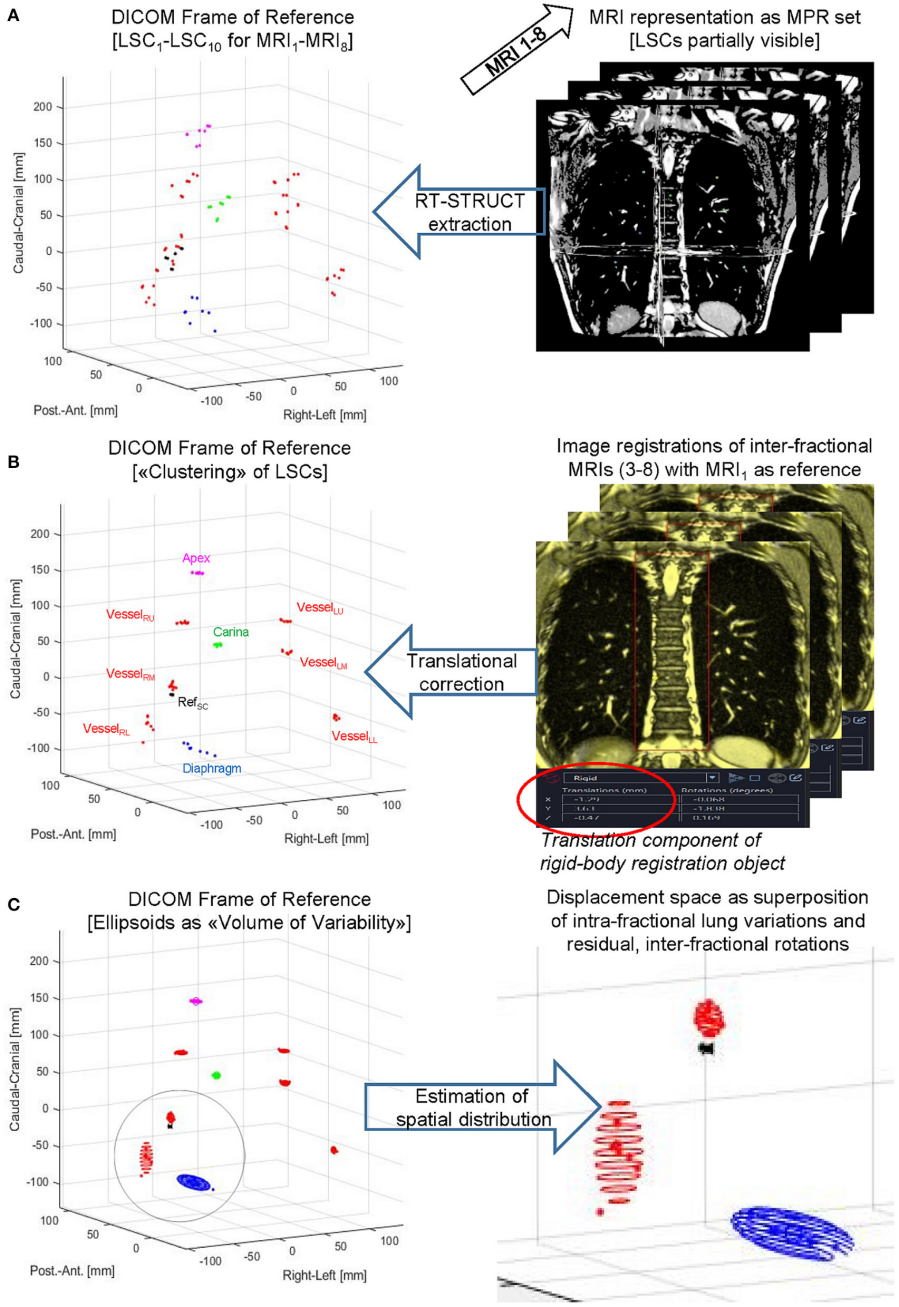
LSC volumes of variability. Principal steps of displaying, processing, and analyzing a lung structure metric for a subject-specific set of eight MRIs acquired for eDIBH or HFPV. Top to bottom: LSCs were contoured as RT-STRUCT sets in Velocity^®^ **(A)** followed by DICOM export/import into MatLab to enable visualization in the common DICOM Frame of Reference. Subsequently **(B)** translation corrections determined by rigid-body image registrations were applied to the LSC distributions identified on left side graph, and **(C)** rotational ellipsoids to approximate their spatial distributions were calculated as described in the text.

Second, as shown schematically in Figure 3, we evaluated (i) the distributions in each subject after corrections for interfractional shifts due to repositioning and (ii) intrafractional differences due to different lung volumes during the consecutive eDIBH or HFPV MRI acquisitions. The analysis consisted of three steps.

(A) After contouring, the spatial distributions of all 10 LSCs were determined in the DICOM reference system of all 16 MRIs: 8 × eDIBH and 8 × HFPV, without positioning correction.
(B) Interfractional shifts were described as rigid-body transformations. These were treated in Velocity^®^ as pairwise image registrations with respect to the reference point Ref_SC_ on the spine between the first MRI of visit 1 (MRI_1_) as reference and the following MRIs from visits 2 to 4 (MRI_3_ to MRI_8_). The transformation between MRI_1_ and the second MRI of visit 1 (MRI_2_) was assumed to be negligible. In the other registrations, the MRIs were superimposed in such a way that the spinal column structure was optimally covered. The resulting six transformation matrices were then exported as DICOM registration objects and imported into MatLab. In order to simulate the 3D positioning offset correction applied in Gantry-2 before each irradiation fraction, only the translation components of the transformation were included in the analysis. These interfractional translation corrections were applied to the corresponding spatial distributions of the LSCs given in (A). For each subject and method, the resulting LSC distributions in Cartesian DICOM space yield clusters of position variabilities.
(C) The standard deviations about the mean positions of each LSC-specific cluster yielded a 95% CL ellipsoid, the volume of which, denoted “volume of variability” (VolOfVar), represented an empirical measure of spatial variability. Division of the VolOfVar by the mean subject lung volume delivered a fractional volume of variability. As for the radial distances, the logarithm of the ratio of fractional variations between the two methods for each subject and lung structure yielded a subject-specific comparison of variability.

## Results

### Breath-Hold Duration and Subject Acceptance

#### Subjective Acceptance

On a scale ranging between 0 and 10, where 0 was the best rating, the subjects rated home-based training as being “feasible”: 2.2 ± 2.1, as they also rated eDIBH and HFPV methods: 1.4 ± 1.1 and 2.2 ± 2.5, respectively. Both methods were also rated “tolerable” for patients: 1.8 ± 2.2 and 2.5 ± 2.7, respectively. Wilcoxon signed rank tests evidenced no difference between the two methods in neither “feasibility” nor “tolerability.” However, 14 of the 21 subjects preferred eDIBH to HFPV; an experience of “less discomfort” during eDIBH was named as the most frequent reason.

#### Effect of Breath-Hold Training in Sitting Position Over Time

**Figure 4:**
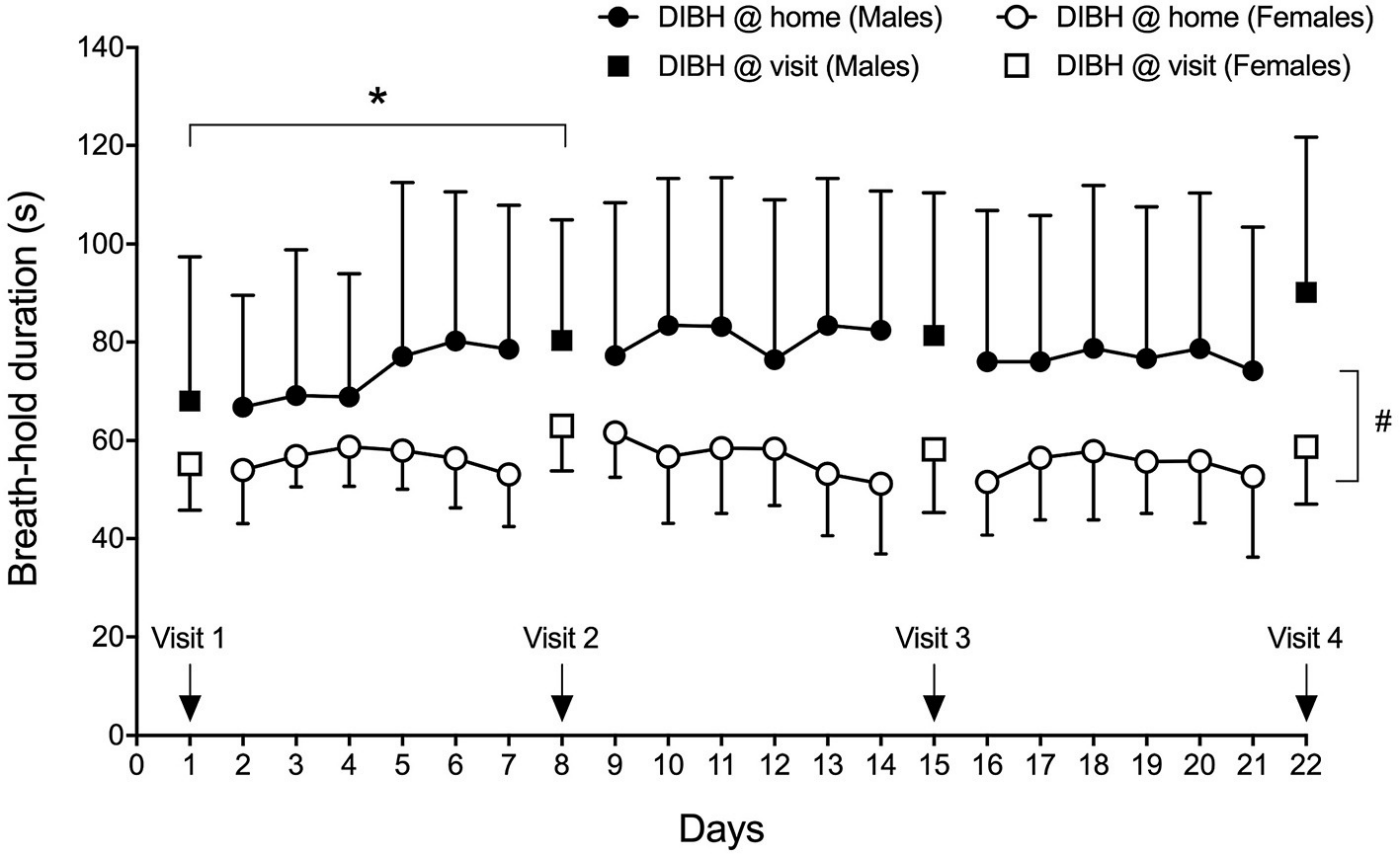
Sex-specific group mean maximal DIBH durations (± standard deviation) at home and at visits. ^*^p < 0.05; #*p* = 0.010.

Breath-hold training at home revealed no change in breath-hold duration in sitting subjects across 18 training days and 4 visits (Figure 4), while a significant effect of time was detected in breath-hold durations assessed at visits (*p* = 0.035), despite breath-hold durations at visits being similar to those assessed at home at these specific time-points (*p* = 0.444). The difference was located between visit 1 and visit 2 (*p* = 0.034). Three deep breaths prior to a maximal DIBH significantly increased breath-hold duration at home in males (18-day average without 77 ± 28 s and with three deep breaths 120 ± 46 s; *p* < 0.001) and females (from 56 ± 9 s to 84 ± 18 s; *p* < 0.001). Also during visits, 3 deep breaths increased breath-hold duration in males (average of four visits without deep breaths: 80 ± 26 s, average of four visits with three deep breaths: 111 ± 38 s, *p* < 0.001) and in females (from 59 ± 7 s to 76 ± 11 s; *p* < 0.001). A significant time-effect was detected that was located *post-hoc* between Visit 1 and Visit 2. A significant sex effect was detected with males having longer breath-hold durations during home trainings (*p* = 0.010) as well as at visits (*p* = 0.038).

#### Breath-Hold Duration Before MRI Acquisition (eDIBH) in Lying Position

**Figure 5:**
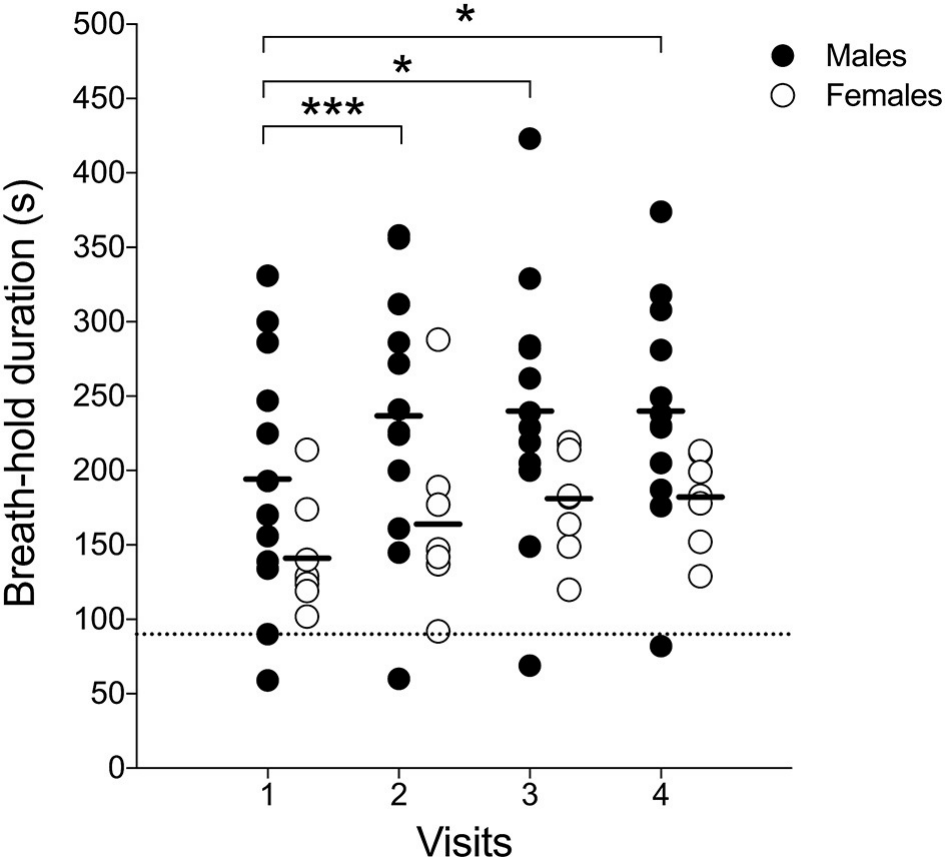
Individual subject's maximal-enhanced deep-inspiration breath hold (eDIBH) duration; black line: group average, dashed line: 90 s duration. ^*^p < 0.01; ^***^p < 0.001.

**Figure 6:**
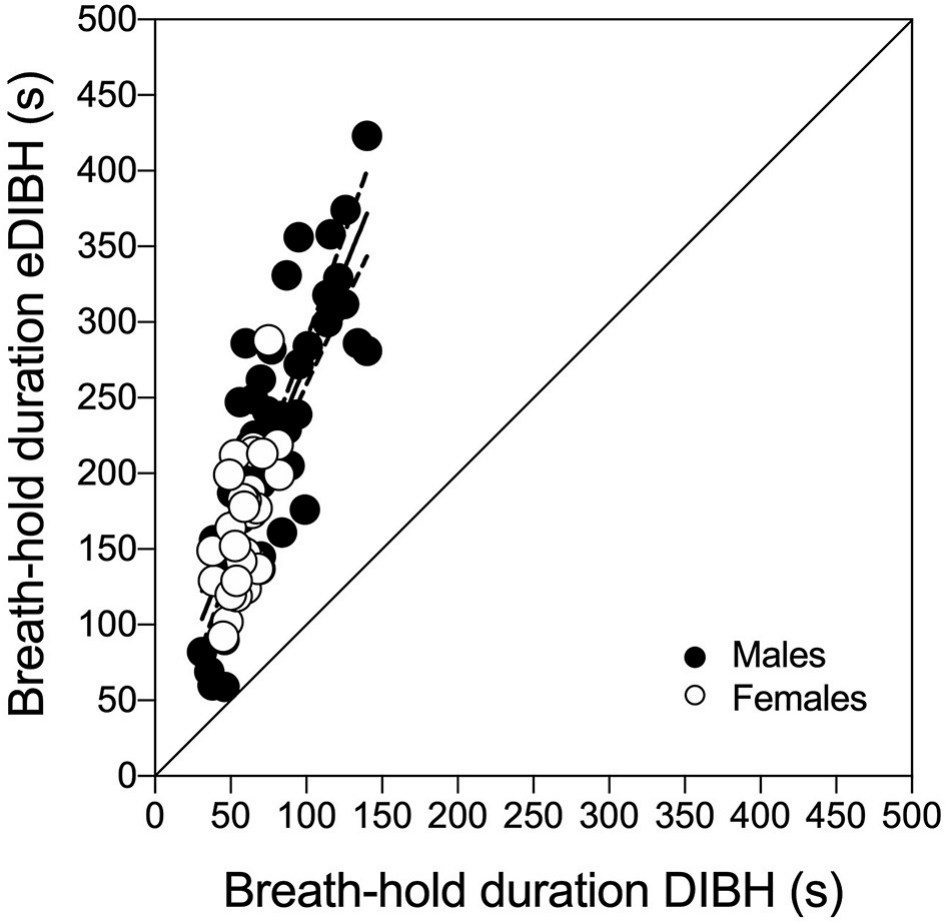
Individual subject's maximal deep-inspiration breath hold (DIBH) durations out of resting breathing in sitting position compared with maximal-enhanced deep-inspiration breath hold (eDIBH) duration in lying position at all visits. Best-fit line with 95% confidence bands.

Enhanced deep-inspiration breath hold duration assessed prior to the MRI acquisition, i.e., eDIBH duration, was significantly lower in visit 1 than in the following visits in the entire group. However, only in males, these differences were present without significant differences between visits in females (Figure 5). Individual breath-hold durations show, that all but one subject reached the required 90 s eDIBH duration at all four visits. DIBH durations assessed in sitting position at home were significantly correlated with eDIBH durations in lying position assessed prior to MRI acquisitions (Figure 6). The correlation coefficients for the different visits were similar: visit 1: *r*^2^ = 0.520, *p* < 0.001; visit 2: *r*^2^ = 0.392, *p* = 0.003; visit 3: *r*^2^ = 0.637, *p* < 0.001; visit 4: *r*^2^ = 0.567, *p* < 0.001. Similarly, sitting breath-hold durations at visits were significantly correlated with eDIBH durations; *r*^2^ = 0.672, *p* < 0.001.

### Lung Volume Analysis

#### Full Inspiration (Forced Vital Capacity)—Changes Within and Between Days

Across visits, measures of FVC did not differ significantly: visit 1: 5.05 ± 1.25 L; visit 2: 5.06 ± 1.24 L; visit 3: 5.05 ± 1.24 L; visit 4: 5.07 ± 1.26 L; *p* = 0.42. However, a sex effect was detected with males having a larger FVC than females (average across four visits in males: 5.93 ± 0.89 L; in females: 3.89 ± 0.37 L, *p* < 0.001). Also, FVC correlated significantly with eDIBH duration (*r*^2^ = 0.306; *p* < 0.001) although—when sexes were analyzed separately—a significant correlation was present in males only (*r*^2^ = 0.279; *p* < 0.001).

To determine FVC according to ATS/ERS criteria (31), an average number of 5 ± 1 attempts (range 3–8) was necessary to achieve maximal values on visit 1 while on visit 4 an average of 3 ± 0 attempts (range 3–4) was needed. On visit 1, all but two subjects reached maximal values at the fourth attempt at the latest, while on visit 4, all but one subject reached maximal values at or prior to the third attempt. Maximal within-subject differences between selected best values ranged between 0.3 and 3.6%.

#### Lung Volumes

The segmentation algorithm yielded the lung volumes presented in Table 2. In addition, their intrafractional variations for each method at each visit were derived.

**Table 2:** All data presented in [l] as mean ± SD.

	**Visit 1**	**Visit 2**	**Visit 3**	**Visit 4**
	**Lung volumes over course of visits [l]**			
eDIBH men	6.40 ± 1.44	6.58 ± 1.20	6.58 ± 1.22	6.59 ± 1.12
eDIBH women	4.61 ± 0.54	4.51 ± 0.78	4.39 ± 0.95	4.38 ± 0.90
HFPV men	5.48 ± 1.41	5.24 ± 1.18	5.67 ± 1.51	5.29 ± 1.73
HFPV women	3.20 ± 0.72	3.48 ± 0.94	3.81 ± 0.74	3.74 ± 0.51
	**Intra-fractional variation of lung volumes [l]**			
eDIBH	0.160 ± 0.256	0.153 ± 0.364	0.056 ± 0.255	0.020 ± 0.285
HFPV	−0.108 ± 0.640	−0.056 ± 0.617	−0.176 ± 0.542	−0.001 ± 0.291

*Top: the means and standard deviations of lung volumes over the course of four visits as determined by gender for eDIBH and HFPV; bottom: the means and standard deviations of the differences between successive measurements of lung volumes for each method at each visit*.

As indicated in Table 2, an unbalanced two-way ANOVA showed no significant variation over visits, *p* < 0.88, but a significant difference between methods, *p* < 0.0001. Further ANOVAs yielded for neither method a significant variation over visit, *p* < 0.99 for eDIBH and *p* < 0.48 for HFPV, but significant difference between sexes, *p* < 0.00001, in both cases. The average difference between males and females for lung volume as measured by eDIBH was 2 L, as for FVC.

In neither of the ANOVAs of the intrafractional variations was there a significant difference over the course of the four visits: *p* < 0.32 for eDIBH and *p* < 0.77 for HFPV. The standard deviations of the intrafractional variations are with the exception of the last visit twice as large for HFPV as for eDIBH and smaller than the lung volume standard deviations given in the upper part of Table 2.

#### Variation of Lung Volume Over Time for eDIBH

**Figure 7:**
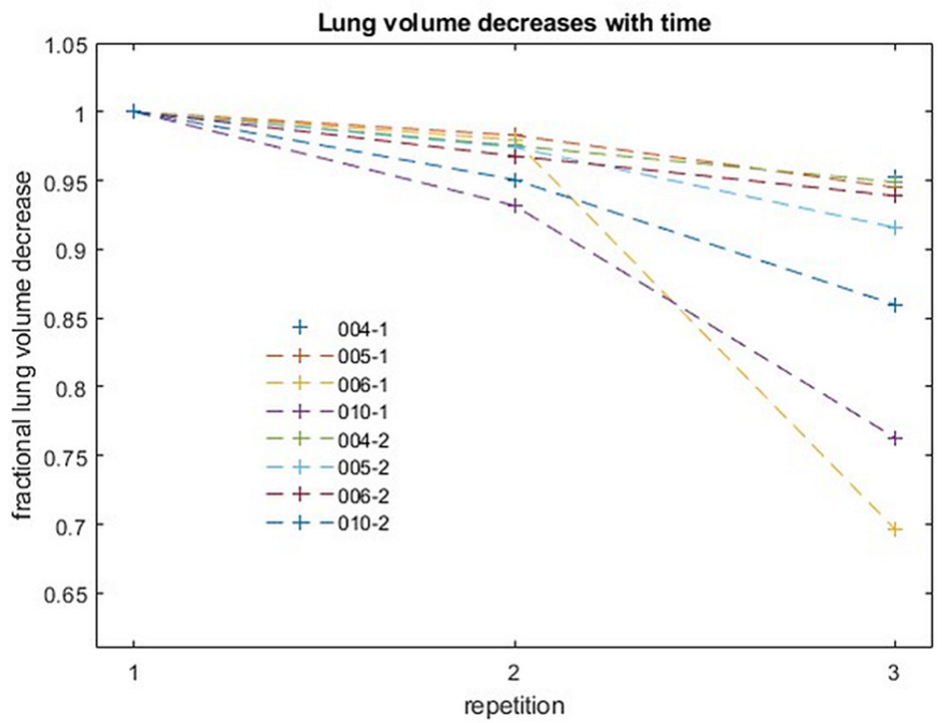
Fractional decreases of lung volume with repetition for four subjects. Each acquisition lasted 70 s. Successive acquisitions were performed with an interruption of a few seconds for restarting the MR sequence.

Analysis of the variation of intrabreath-hold lung volumes for the four subjects most capable of long breath-hold durations (Figure 7) showed that the decrease in lung volume over the course of the first 70 s acquisition was 5% or less in each of the four subjects. The more pronounced decrease in subject 6 (006-1) was rather due to an image artifact than to a physiological effect. The measured, maximum 5% decrease in lung volume over a period corresponding to the radiation time of a single field was thus below the 10% threshold, which was determined to be a sufficient determination accuracy for the automated calculation of lung volumes.

### Lung Topography Analysis

#### Intrafractional Displacement of Lung Structure Metrics

The first analysis of lung structure metric evaluates the positional variability of the lung structures *via* the relative errors of radial distances *r*_i_ between Ref_SC_ and LSC_i_, i.e., the quotient of the standard deviation and the mean value of the LSC_i_ distributions over all subjects. The analysis yielded no significant correlation between the relative errors of lung structures and the relative errors of the computed lung volumes. They can therefore serve as intrinsic measures of variability.

As given in Table 3, the medians and maxima for almost all structures are consistently less for eDIBH than for HFPV. In addition, the Wilcoxon signed rank test yielded significant differences between methods for the diaphragm, the carina, and vessels in the lower region of both lungs, Vessel_LL_ and Vessel_RL_, and one in the left mid-lung section, Vessel_LM_. This indicates a significantly lower positional variability for eDIBH than for HFPV.

**Table 3:** Median and maximum relative error [%] of radial distances between reference (Ref_SC_) and selected anatomical structures (LSC_i_), including significance of difference between corresponding distributions of eDIBH and HFPV according to Wilcoxon signed rank test.

**Relative errors of radial distances to reference/significance of difference**					
**LSC**	**eDIBH**		**HFPV**		***p***
	**Median**	**Maximum**	**Median**	**Maximum**	
	**[%]**	**[%]**	**[%]**	**[%]**	
Apex	0.7980	1.3164	0.7876	3.2929	0.167
Carina	2.4417	3.8687	3.3148	7.1217	0.014
Diaphragm	2.6896	9.3390	7.7671	25.0294	0.001
Vessel_LU_	1.1106	2.0416	1.2444	3.8462	0.218
Vessel_LM_	1.5520	3.0509	2.3186	3.5505	0.025
Vessel_LL_	1.7521	7.5847	3.7943	13.1986	0.010
Vessel_RU_	1.4460	1.9090	1.4487	4.3269	0.314
Vessel_RM_	2.2613	7.0645	2.3931	6.3809	0.247
Vessel_RL_	3.6881	16.3864	7.7566	21.1520	0.014

**Figure 8:**
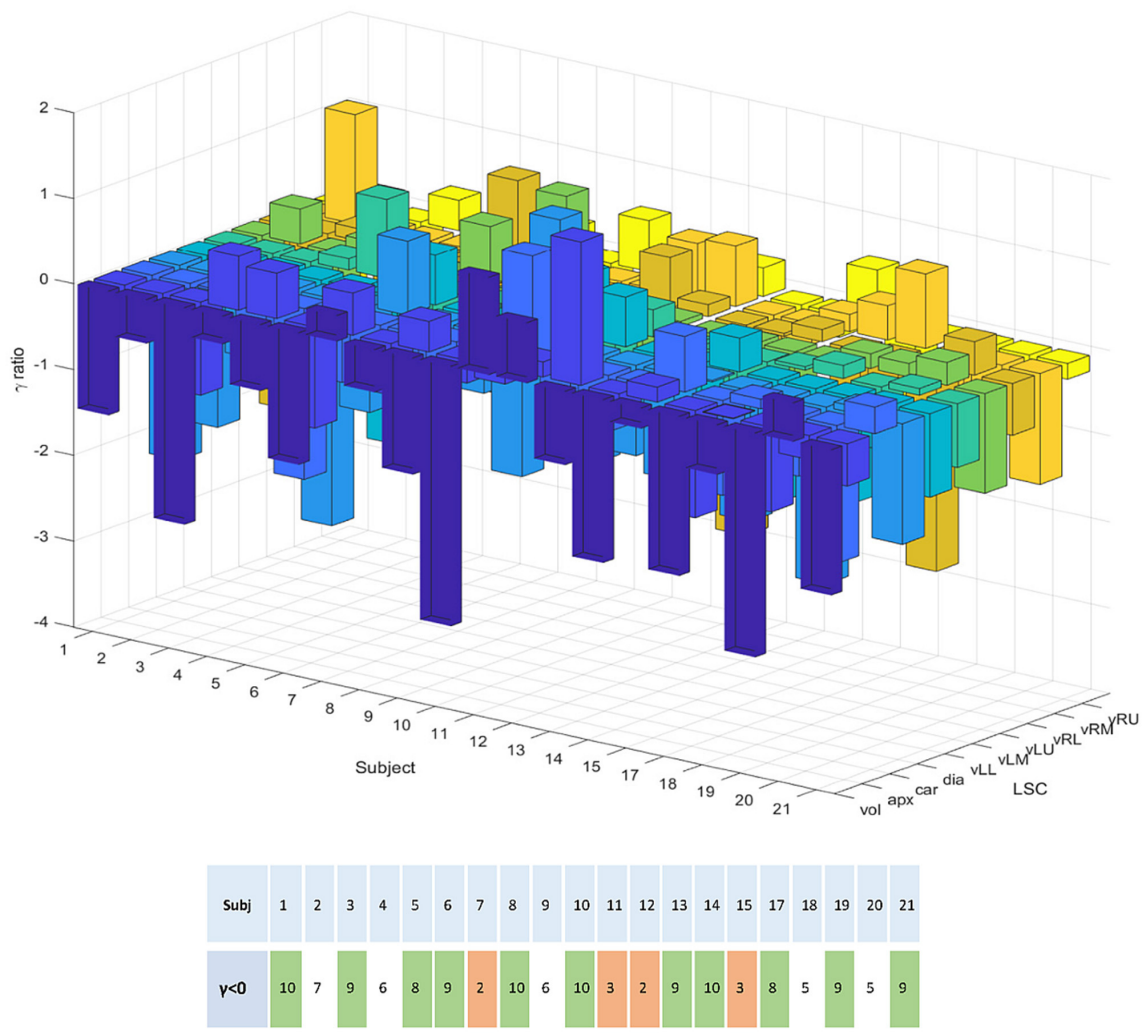
The 3D bar plot displays the logarithm of the ratio, denoted γ, of relative errors in lung structure radial distances and volumes between the motion mitigation methods, eDIBH and HFPV, for each subject, and lung structure or volume. The lower table assigns to each subject the number of values for which the γ ratio is negative, indicating less variability with eDIBH. Green indicates numbers >7, orange those <4. White indicates intermediate numbers.

Confirmation of this result on a subject basis was established by plotting the logarithm of the ratio of relative errors between eDIBH and HFPV, denoted as γ ratio, for each subject and LSC. This yielded the three-dimensional bar plot in the upper part of Figure 8. Augmented by the corresponding ratio of lung volumes, it summarizes the relative variability of the two motion mitigation methods. A negative logarithm indicates that the variability is less for eDIBH, a positive value that the variability is less for HFPV.

Of the 200 log ratios, 140, i.e., 70%, show less variability for eDIBH. The subject distribution presented in the resulting table at the bottom of Figure 8 showed in 11 subjects between 8 and 10 γ ratios favoring eDIBH, whereas, four subjects exhibit between 0 and 3 of such ratios. Thus, of the 15 subjects showing a clear distinction, 73% of the subjects show less variability for eDIBH. The distribution in Figure 8 can be modeled by a binomial distribution with the two options eDIBH or HFPV, resulting in a probability of 0.87 for eDIBH. This value indicates that the above estimates of less variability for eDIBH are not arbitrary.

#### Interfractional Displacement of Lung Structure Metrics

**Figure 9:**
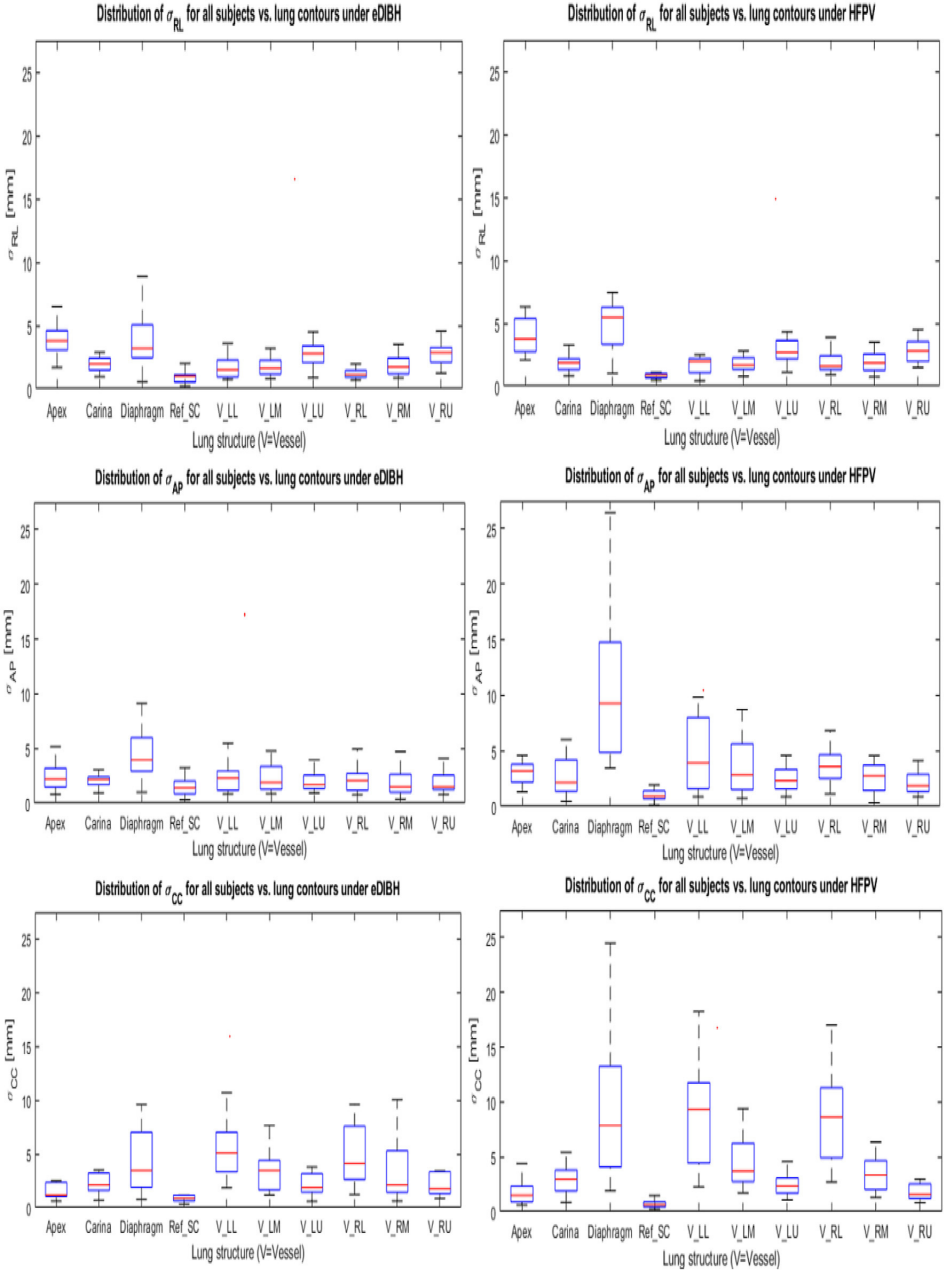
Standard deviations about the mean positions of each LSC-specific cluster in Cartesian coordinates for eDIBH and HFPV (see Section **Measuring Intra- and Interfractional Lung Displacements** and Figure 3). The coordinates are labeled in table notation: RL (top row), AP (middle row), and CC (lower row).

To simulate proton therapy treatment conditions for Gantry-2@PSI, where setup uncertainties are compensated by linear 3D offset movements of the couch, the inter-fractional displacements (contained in the 6 DoF-MRI registration objects) were corrected by applying their 3D translation components to the respective lung structure distributions (see Figure 3). Their remaining spatial uncertainties are thus composed of (i) the interfractional rotational components and (ii) the interbreath-hold variations. Geometrically, rotations around the AP axis result in lateral displacements. Their amount depends on the distance to the rotation center Ref_SC_ in CC direction. Since the spatial shifts of lung structures due to respiratory movements are least pronounced in RL (39), the contribution of uncorrected setup rotations is greatest in the lateral spatial direction. Assuming a CC distance of a pulmonary vessel from Ref_SC_ of 100 mm, an AP rotation of 3° delivers a lateral offset of about 5 mm. A comparison with Figure 9 reveals this value to be a good approximation to the upper limits for the σ_RL_ distributions of Vessel_LU_ and Vessel_RU_ displayed in the top row. Larger RL displacements were measured only for the apex: ~6–7 mm and diaphragm: ~8–10 mm, as these lung structures are farthest from the reference point in the CC direction. There are no significant differences between eDIBH and HFPV regarding RL displacements.

In accordance with the intrafractional displacements discussed in Section **Intra-Fractional Displacement of Lung Structure Metrics**, the most significant differences between eDIBH and HFPV in spatial variability are found in the CC and AP directions for the diaphragm, carina and for the vessels of the lower and middle left lung segment.

Analogous to the relationship between rotational distribution and RL shifts, the contributions of pitch and roll components to the linear shifts of lung structures in the CC and AP direction can be estimated geometrically. Calculations reveal that only about 10–20% of these displacements are caused by the uncorrected rotations about the RL and CC axes, respectively; the variation in lung volumes due to eDIBH or HFPV represent the dominant factor.

This presentation of lung structure variability in Cartesian space concludes with analysis of comparison of the relative volumes of variability: VolOfVar_eDIBH_ and VolOfVar_HFPV_, determined by the ratio between the 95% CL ellipsoid volume and the mean subject lung volume for each subject and lung vessel structure.

**Figure 10:**
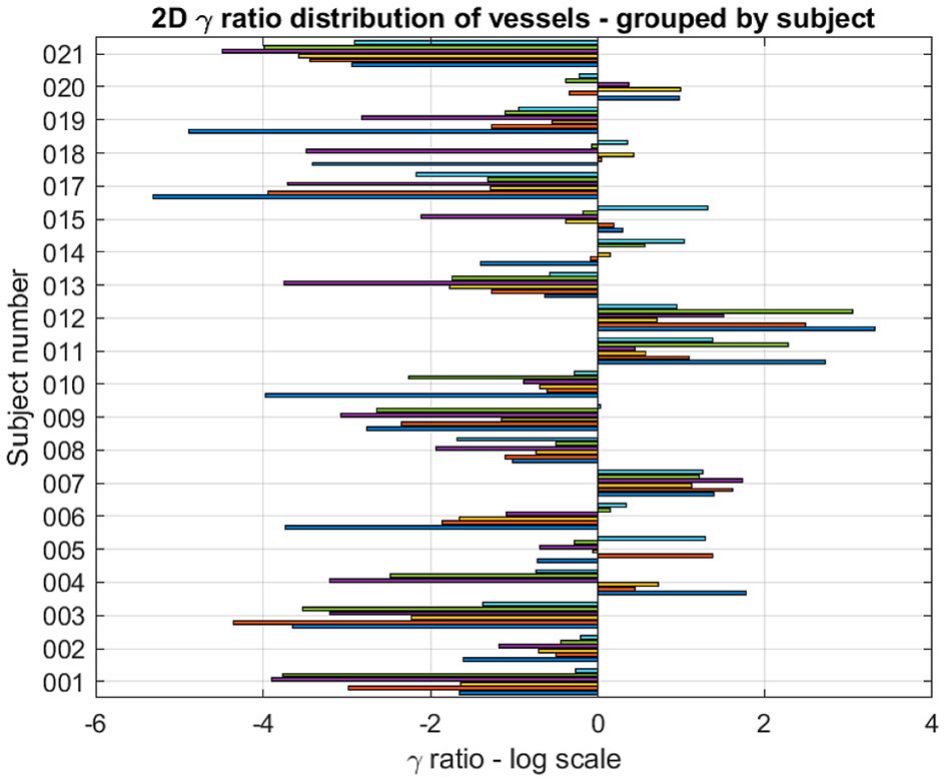
For each subject and lung vessel are plotted the logarithm of the ratio of relative volumes of variability determined from the 95% CL ellipsoids of variability between the eDIBH and HFPV. Negative values indicate lesser variability with eDIBH, positive values lesser variability with HFPV.

The logarithm of the ratio of eDIBH and HFPV relative volumes is shown in Figure 10 as 2D bar plots. Of the 120 ratios which could be determined, 85 showed less variability with eDIBH, i.e., 71%, confirming the analysis of relative errors in radial distances presented earlier. However, only 10 of the 20 subjects showed exclusively less variability with eDIBH, whereas, three showed exclusively less variability with HFPV; the remaining seven subjects exhibited mixed variabilities.

## Discussion

External beam radiation therapy is a highly effective modality in the treatment of lung tumors, yielding similar outcomes as surgery, particularly for early stage non-small-cell lung carcinoma [NSCLC; (40, 41)]. Appropriate respiratory motion management approaches (17), however, are of great importance, especially for PBS-PT (42). As such, breath holding is an effective method to minimize respiratory motion, which is well-established in conventional RT treatments of breast cancer and mediastinal lymphoma (43–46). Its combination with hyperventilation and/or preoxygenation for breast irradiation with photons has already been reported (47).

Several studies investigated the use of DIBH for lung cancer RT (26, 39, 48–50). However, clinical breath-hold implementations in proton therapy of lung tumors are currently predominated by device-assisted breath-hold techniques (17, 51–53). Training or coaching, however, is deemed necessary for patient applications, with autonomous breath holding with or without monitoring being the exception. To the authors' knowledge, however, no reports of practical implementation for patient-controlled DIBH supported by physiological measures (hyperventilation, preoxygenation, breath-hold training) are available for application in proton therapy, and only few studies have related breath-hold duration to treatment delivery time. Indeed, the Particle Therapy Cooperative Group (PTCOG) guidelines (54) for implementing PBS-PT in thoracic malignancies consider DIBH to be a realistic clinical treatment approach. However, they also caution that small highly mobile tumors potentially causing large baseline drifts are a significant risk for a consistent DIBH application. Research and clinical practice in the field of motion management for particle therapy, however, has primarily focused on adapting to motion (rescanning, gating, etc.,) rather than effectively mitigating it by eliminating or reducing patient organ motion, e.g., through breath hold.

Following the assumption that effective motion management starts with mitigation, the change in breath-hold duration with breath-hold training and the use of hyperventilation combined with oxygenation were investigated in the present study with 21 healthy subjects. Using MRI assessment to simulate realistic PT treatment conditions, the newly proposed eDIBH and the established HFPV application for respiratory motion suppression were investigated with respect to lung volume variability and reproducibility of lung topographies. The aim of these measurements was the evaluation of applicability and effectiveness of both methods. Since the use of eDIBH envisages breath-hold training prior to the start of proton therapy, not only the effect on breath-hold duration over the study period was analyzed, but also subjects' acceptance of the eDIBH approach compared to the HFPV method was determined.

The main results of the present study demonstrated that by the use of breath-hold training and DIBH enhanced by prior hyperventilation in hyperoxic conditions, breath-hold durations could be increased from 173 ± 73 s on visit 1 to 217 ± 67 s on visit 4, with maximal eDIBH durations in all but one individual subject reaching an eDIBH duration of ≥90 s already at visit 1. The achieved breath-hold durations were similar or longer than the time required to deliver representative PBS proton treatment fields at PSI's Gantry-2 for typical lung tumors. A recent study using a different method to extend breath-hold duration in pulmonary patients (a majority with stage III lung cancer) reported a mean breath-hold duration of 78 s (range 29–223 s) during conventional RT treatment (55). Thus, the increase in breath-hold duration by eDIBH in the healthy subjects of the present study is likely transferrable to lung cancer patients allowing irradiation of adequate PBS-PT fields. Importantly also, lung volumes determined prior to and during breath holds, did not change over the course of four visits. In addition, an important advantage of eDIBH found in this work results from the fact that subjective tolerance of eDIBH was similar to HFPV and presumed, by study subjects, tolerable for patients. Also, home training was given a good rating. Indeed, of the 18 subjects expressing a distinct preference, 14 (~80%) preferred eDIBH to HFPV. Also, during the study, no adverse events occurred with eDIBH, but one subject was unable to tolerate HFPV.

Detailed analysis showed that lung volumes determined with spirometry and during eDIBH in the MR were larger in males than females. This translated into a tendency for shorter eDIBH duration in females compared to males. Nevertheless, in this female cohort, breath-hold duration was still long enough for a PRT with 141 ± 36 s already on visit 1. The fact that breath-hold duration in both sexes did not change significantly after visit 2 suggests that 1 week of individual home-based breath-hold training with four daily maximal DIBHs, including reassurance of the possibility to extend breath-holds, e.g., by prior deep breathing (56), is sufficient to achieve maximal breath-hold durations. Also, the good correlation found in this study between maximal sitting DIBHs at home and maximal eDIBHs could possibly allow to predict in future patients, already after 1 week of home-based DIBH-training, whether individual patients' breath holds will be of sufficient duration for effective irradiation.

While sufficiently long breath hold durations are essential, it is equally crucial to achieve consistently similar lung volumes and lung topographies over all irradiation days. As for reproducibility of lung volume, no significant difference in FVC and in inter-fractional variability was observed over the course of four visits, with intrafractional deviations being <10% of the standard deviation of uncertainty of the volume measurements. Thus, lung volume was reproducible and stable over the study course, especially also within each visit. During extended breath holds over the duration of three consecutive, identical MRI sequences, however, as tested on a sub-set of subjects, lung volume was found to decrease significantly after the second sequence repetition. This effects results from the ongoing metabolism where burning a mixture of carbohydrate and fat produces a smaller volume of CO_2_ than the volume of O_2_ consumed in a given time period (respiratory quotient <1.0). Nevertheless, such volume reductions stayed below 5% over the first and second MRI sequence, which is less than the uncertainty in intrafractional volume determination.

The advantages of eDIBH became evident in the analysis of lung structure positional variability based on a lung structure metric. Five of nine absolute distances between mobile lung structures and a static reference structure at the spine showed, averaged over all subjects, significantly less variability of relative error for eDIBH than for HFPV. These were the carina, diaphragm, and the vessels in the left and right lower lung segment and in the left-middle lung region. The introduction of a subject- and lung structure-specific measure to determine distance variability between eDIBH and HFPV defined by the logarithmic ratio of their relative errors indicated that eDIBH exhibited at least a 70% advantage compared to HFPV. This result was derived from the total number of ratios calculated, as well as by the overall number of subjects showing less variability for eDIBH.

In addition, an approximation of the intrafractional, spatial distribution of the 10 lung structures across all MRIs of each subject provided —after correction of the interfractional translations and separately for eDIBH and HFPV—their individual so-called Volume of Variability. In analogy to the variability of distances, the logarithmic ratio of VolOfVar_eDIBH_, and VolOfVar_HFPV_ confirmed that the spatial variability for all investigated pulmonary vessels is smaller under eDIBH than for HFPV in 71% of cases. The distributions of positional standard deviations of the 10 lung structures in all three Cartesian dimensions showed that standard deviations are least for the reference structures on which the registrations were based, and most for the diaphragm and for vessels in the lower left and right lung segment. Their magnitude is higher by a factor of 3 for the diaphragm using HFPV than for eDIBH and by a factor of 2 for Vessel_LL_ and Vessel_RL_ with differences between the methods being most apparent in the anterior-posterior and cranial-caudal directions.

In summary, the present results can be interpreted in three ways. Physically, the eDIBH approach has significant and clear advantages over the HFPV method with respect to local reproducibility and stability of lung conditions over time. In practical application, both approaches are feasible under irradiation conditions with proton therapy, whereby both the subjective preference of the study participants and the lower resource requirements speak in favor of eDIBH. Physiologically, the results suggest that the eDIBH procedure could benefit patients that need to achieve a breath-hold duration of ≥60–90 s for irradiation therapy. Although, it can be expected that such durations can be achieved in cancer patients without lung morbidity and despite such breath-hold durations having been reported in lung cancer patients (55), further investigations are needed to test the extent to which compromised lung function influences overall breath-hold performance.

In articles related to radiotherapy, average breath-hold durations of around 22 s are reported for breath holds starting from resting breathing (47). This is 2- to 3-fold less than breath holds reported in the present study but in those studies, no or much less emphasis was put on achieving maximal lung volumes at the start of those breath holds. Thus, although the present results are very promising, more research is needed in lung tumor patients in order to ascertain the benefit of the present eDIBH procedure in this patient population.

## Conclusions

Both, eDIBH, and HFPV were well-tolerated and eDIBH duration was long enough to allow potential PRT. Variability in lung volume and anatomical position of lung structures is smaller with eDIBH. Also, if given the opportunity to choose, subjects prefer eDIBH. Thus, eDIBH is a very promising tool for lung tumor therapy with PRT, and further investigation of its applicability in patients is warranted.

## Data Availability Statement

Raw data supporting the conclusions of this article are provided by the authors upon reasonable request.

## Ethics Statement

The studies involving human participants were reviewed and approved by Cantonal Ethics Committee North West and Central Switzerland. The patients/participants provided their written informed consent to participate in this study.

## Author Contributions

FE, PE, MW, DW, and CS contributed to the conception and design of the work. FE, MW, and CG were involved in the acquisition of the data. FE, JM, MW, CG, PE, and CS analyzed and interpreted the data. FE, JM, PE, CS, and AL drafted the manuscript. All authors contributed to the manuscript and approved the submitted version.

## Conflict of Interest

The authors declare that the research was conducted in the absence of any commercial or financial relationships that could be construed as a potential conflict of interest.

## References

[1] Mayles P Nahum A Rosenwald JC (editors). Handbook of Radiotherapy Physics: Theory and Practice. 1st ed. Boca Raton, FL: CRC Press (2007). 10.1201/9781420012026

[2] International Commission on Radiation Units and Measurements. ICRU Report 50: Prescribing, Recording, and Reporting Photon Beam Therapy. Washington, DC: International Commission on Radiation Units and Measurements (1993).

[3] International Commission on Radiation Units and Measurements. ICRU Report 62, Prescribing, Recording, and Reporting Photon Beam Therapy (Supplement to ICRU Report 50). Bethesda, MD: International Commission on Radiation Units and Measurements (1999).

[4] International commission on radiation units and measurements. ICRU report 78, prescribing, recording, and reporting proton-beam therapy. J Int Comm Radiat Unit Meas. (2007) 7:11–150. 10.1093/jicru/ndn001

[5] Durante M Paganetti H. Nuclear physics in particle therapy: a review. Rep Prog Phys. (2016) 79:096702. 10.1088/0034-4885/79/9/096702 27540827

[6] Palta J Yeung D. Precision and uncertainties in proton therapy for nonmoving targets. In: Paganetti H, editor. Proton Therapy Physics. 1st ed. Boca Raton, FL: CRC Press (2012). p. 413–33. 10.1201/b11448-14

[7] Engelsman M Bert C. Precision and uncertainties in proton therapy for moving targets. In: Paganetti H, editor. Proton Therapy Physics. 1st ed. Boca Raton, FL: CRC Press (2012). p. 435–59. 10.1201/b11448-15

[8] Bert C Durante M. Motion in radiotherapy: particle therapy. Phys Med Biol. (2011) 56:R113–44. 10.1088/0031-9155/56/16/R01 21775795

[9] Bert C Herfarth K. Management of organ motion in scanned ion beam therapy. Radiat Oncol. (2017) 12:170. 10.1186/s13014-017-0911-z 29110693 PMC5674859

[10] Bert C Grözinger SO Rietzel E. Quantification of interplay effects of scanned particle beams and moving targets. Phys Med Biol. (2008) 53:2253–65. 10.1088/0031-9155/53/9/003 18401063

[11] Phillips MH Pedroni E Blattmann H Boehringer T Coray A Scheib S. Effects of respiratory motion on dose uniformity with a charged particle scanning method. Phys Med Biol. (1992) 37:223–34. 10.1088/0031-9155/37/1/016 1311106

[12] Wink KCJ Roelofs E Solberg T Lin L Simone CB II Jakobi A. Particle therapy for non-small cell lung tumors: where do we stand? A systematic review of the literature. Front Oncol. (2014) 4:292. 10.3389/fonc.2014.00292 25401087 PMC4212620

[13] Grassberger C Dowdell S Sharp G Paganetti H. Motion mitigation for lung cancer patients treated with active scanning proton therapy. Med Phys. (2015) 42:2462–9. 10.1118/1.4916662 25979039 PMC4409624

[14] Bert C Rietzel E. 4D treatment planning for scanned ion beams. Radiat Oncol. (2007) 2:24. 10.1186/1748-717X-2-24 17608919 PMC1952066

[15] Dong L Yang J Zhang Y. Image processing in adaptive radiotherapy. In: Brock KK, editor. Image Processing in Radiation Therapy. 1st ed. Boca Raton, FL: CRC Press (2013). p. 1–20.

[16] Kubiak T. Particle therapy of moving targets-the strategies for tumour motion monitoring and moving targets irradiation. Br J Radiol. (2016) 89:20150275. 10.1259/bjr.20150275 27376637 PMC5124789

[17] Keall PJ Mageras GS Balter JM Emery RS Forster KM Jiang SB. The management of respiratory motion in radiation oncology report of AAPM Task Group 76. Med Phys. (2006) 33:3874–900. 10.1118/1.2349696 17089851

[18] Lomax AJ Böhringer T Bolsi A Coray D Emert F Goitein G. Treatment planning and verification of proton therapy using spot scanning: initial experiences. Med Phys. (2004) 31:3150–7. 10.1118/1.1779371 15587667

[19] Lomax AJ. Intensity modulated proton therapy and its sensitivity to treatment uncertainties 1: the potential effects of calculational uncertainties. Phys Med Biol. (2008) 53:1027–42. 10.1088/0031-9155/53/4/014 18263956

[20] Knopf AC Stützer K Richter C Rucinski A da Silva J Phillips J. Required transition from research to clinical application: report on the 4D treatment planning workshops 2014 and 2015. Phys Med. (2016) 32:874–82. 10.1016/j.ejmp.2016.05.064 27328991

[21] Trnkova P Knaeusl B Actis O Bert C Biegun AK Boehlen TT. Clinical implementations of 4D pencil beam scanned particle therapy: report on the 4D treatment planning workshop 2016 and 2017. Phys Med-Eur J Med Phys. (2018) 54:121–30. 10.1016/j.ejmp.2018.10.002 30337001

[22] Riboldi M Orecchia R Baroni G. Real-time tumour tracking in particle therapy: technological developments and future perspectives. Lancet Oncol. (2012) 13:e383–91. 10.1016/S1470-2045(12)70243-7 22935238

[23] De Ruysscher D Sterpin E Haustermans K Depuydt T. Tumour movement in proton therapy: solutions and remaining questions: a review. Cancers. (2015) 7:1143–53. 10.3390/cancers7030829 26132317 PMC4586762

[24] Lu HM Brett R Sharp G Safai S Jiang S Flanz J. A respiratory-gated treatment system for proton therapy. Med Phys. (2007) 34:3273–8. 10.1118/1.2756602 17879790

[25] Kanehira T Matsuura T Takao S Matsuzaki Y Fujii Y Fujii T. Impact of real-time image gating on spot scanning proton therapy for lung tumors: a simulation study. Int J Radiat Oncol Biol Phy. (2017) 97:173–81. 10.1016/j.ijrobp.2016.09.027 27856039

[26] Boda-Heggemann J Knopf AC Simeonova-Chergou A Wertz H Stieler F Jahnke A. Deep inspiration breath hold-based radiation therapy: a clinical review. Int J Radiat Oncol Biol Phy. (2016) 94:478–92. 10.1016/j.ijrobp.2015.11.049 26867877

[27] Péguret N Ozsahin M Zeverino M Belmondo B Durham AD Lovis A. Apnea-like suppression of respiratory motion: first evaluation in radiotherapy. Radiother Oncol. (2016) 118:220–6. 10.1016/j.radonc.2015.10.011 26979264

[28] Zhang Y Huth I Weber DC Lomax AJ. A statistical comparison of motion mitigation performances and robustness of various pencil beam scanned proton systems for liver tumour treatments. Radiother Oncol. (2018) 128:182–8. 10.1016/j.radonc.2018.01.019 29459153

[29] Flume PA Eldridge FL Edwards LJ Mattison LE. Relief of the 'air hunger' of breathholding. A role for pulmonary stretch receptors. Respirat Physiol. (1996) 103:221–32. 10.1016/0034-5687(95)00094-1 8738898

[30] Waurick S Rammelt S Rassler B Teller H. Breathing–homeostatic function and voluntary motor activity. Pflugers Archiv. (1996) 432(Suppl. 3):R120–6. 8994553

[31] Miller MR Hankinson J Brusasco V Burgos F Casaburi R Coates A. ATS/ERS Task Force. Standardisation of spirometry. Eur Respir J. (2005) 26:319–38. 10.1183/09031936.05.00034805 16055882

[32] Wanger J Clausen JL Coates A Pedersen OF Brusasco V Burgos F. Standardisation of the measurement of lung volumes. Eur Respir J. (2005) 26:511–22. 10.1183/09031936.05.00035005 16135736

[33] Fowler WS. Breaking point of breath-holding. J Appl Physiol. (1954) 6:539–45. 10.1152/jappl.1954.6.9.539 13152037

[34] Flume PA Eldridge FL Edwards LJ Houser LM. The fowler breathholding study revisited: continuous rating of respiratory sensation. Respirat Physiol. (1994) 95:53–66. 10.1016/0034-5687(94)90047-7 8153453

[35] Herzog M Sucec J Van Diest I Van den Bergh O Chan P-YS Davenport P. Reduced neural gating of respiratory sensations is associated with increased dyspnoea perception. Eur Respir J. (2018) 5:1800559. 10.1183/13993003.00559-2018 29773692

[36] Bain AR Drvis I Dujic Z MacLeod DB Ainslie PN. Physiology of static breath holding in elite apneists. Exp Physiol. (2018) 103:635–51. 10.1113/EP086269 29512224

[37] Parkes MJ. Breath-holding and its breakpoint. Exp Physiol. (2006) 91:1–15. 10.1113/expphysiol.2005.031625 16272264

[38] Parkes MJ. The limits of breath holding. Sci Am. (2012) 306:74–9. 10.1038/scientificamerican0412-74 22486121

[39] Josipovic M Persson GF Dueck J Bangsgaard JP Westman G Specht L. Geometric uncertainties in voluntary deep inspiration breath hold radiotherapy for locally advanced lung cancer. Radiother Oncol. (2016) 118:510–4. 10.1016/j.radonc.2015.11.004 26631647

[40] Baker S Dahele M Lagerwaard FJ Senan S. A critical review of recent developments in radiotherapy for non-small cell lung cancer. Radiat Oncol. (2016) 11:115. 10.1186/s13014-016-0693-8 27600665 PMC5012092

[41] Harada H Murayama S. Proton beam therapy in non-small cell lung cancer: state of the art. Lung Cancer. (2017) 8:141–5. 10.2147/LCTT.S117647 28883747 PMC5574682

[42] Vyfhuis M Onyeuku N Diwanji T Mossahebi S Amin NP Badiyan SN. Advances in proton therapy in lung cancer. Ther Adv Respir Dis. (2018) 12:1–16. 10.1177/1753466618783878 30014783 PMC6050808

[43] Latty D Stuart KE Wang W Ahern V. Review of deep inspiration breath-hold techniques for the treatment of breast cancer. J Med Radiat Sci. (2015) 62:74–81. 10.1002/jmrs.96 26229670 PMC4364809

[44] Bergom C Currey A Desai N Tai A Strauss JB. Deep inspiration breath hold: techniques and advantages for cardiac sparing during breast cancer irradiation. Front Oncol. (2018) 8:87. 10.3389/fonc.2018.00087 29670854 PMC5893752

[45] Aznar MC Maraldo MV Schut DA Lundemann M Brodin NP Vogelius IR. Minimizing late effects for patients with mediastinal Hodgkin lymphoma: deep inspiration breath-hold, IMRT, or both? Int J Radiat Oncol Biol Phys. (2015) 92:169–74. 10.1016/j.ijrobp.2015.01.013 25754634

[46] Baues C Marnitz S Engert A Baus W Jablonska K Fogliata A. Proton versus photon deep inspiration breath hold technique in patients with hodgkin lymphoma and mediastinal radiation : a planning comparison of deep inspiration breath hold intensity modulation radiotherapy and intensity modulated proton therapy. Radiat Oncol. (2018) 13:122. 10.1186/s13014-018-1066-2 29970105 PMC6029162

[47] Roth J Engenhart-Cabillic R Eberhardt L Timmesfeld N Strassmann G. Preoxygenated hyperventilated hypocapnic apnea-induced radiation (PHAIR) in breast cancer patients. Radiother Oncol. (2011) 100:231–5. 10.1016/j.radonc.2011.02.017 21497926

[48] Wong JW Sharpe MB Jaffray DA Kini VR Robertson JM Stromberg JS. The use of active breathing control (ABC) to reduce margin for breathing motion. Int J Radiat Oncol Biol Phys. (1999) 44:911–9. 10.1016/S0360-3016(99)00056-5 10386650

[49] Hanley J Debois MM Mah D Mageras GS Raben A Rosenzweig K. Deep inspiration breath-hold technique for lung tumors: the potential value of target immobilization and reduced lung density in dose escalation. Int J Radiat Oncol Biol Phys. (1999) 45:603–11. 10.1016/S0360-3016(99)00154-6 10524412

[50] Mah D Hanley J Rosenzweig KE Yorke E Braban L Ling CC. Technical aspects of the deep inspiration breath-hold technique in the treatment of thoracic cancer. Int J Radiat Oncol Biol Phys. (2000) 48:1175–85. 10.1016/S0360-3016(00)00747-1 11072177

[51] Remouchamps VM Letts N Vicini FA Sharpe MB Kestin LL Chen PY. Initial clinical experience with moderate deep-inspiration breath hold using an active breathing control device in the treatment of patients with left-sided breast cancer using external beam radiation therapy. Int J Radiat Oncol Biol Phys. (2003) 56:704–15. 10.1016/S0360-3016(03)00010-5 12788176

[52] Giraud P Morvan E Claude L Mornex F Le Pechoux C Bachaud JM. Respiratory gating techniques for optimization of lung cancer radiotherapy. J Thorac Oncol. (2011) 6:2058–68. 10.1097/JTO.0b013e3182307ec2 22052228

[53] Mazars P Garcia R. 12 Study of the anatomy position reproducibility during deep inspiration breath hold. Phys Med. (2018) 56:45–6. 10.1016/j.ejmp.2018.09.09410524412

[54] Chang JY Zhang X Knopf A Li H Mori S Dong L. Consensus guidelines for implementing pencil-beam scanning proton therapy for thoracic malignancies on behalf of the PTCOG thoracic and lymphoma subcommittee. Int J Radiat Oncol Biol Phys. (2017) 99:41–50. 10.1016/j.ijrobp.2017.05.014 28816159

[55] Peeters S Vaassen F Hazelaar C Vaniqui A Rousch E Tissen D. Visually guided inspiration breath-hold facilitated with nasal high flow therapy in locally advanced lung cancer. Acta Oncol. (2020). 10.1080/0284186X.2020.1856408. [Epub ahead of print]. 33295823

[56] Klocke FJ Rahn H. Breath holding after breathing of oxygen. J Appl Physiol. (1959) 14:689–93. 10.1152/jappl.1959.14.5.689 14409935

